# HERO: Hybrid Effortless Resilient Operation Stations for Flash Flood Early Warning Systems

**DOI:** 10.3390/s22114108

**Published:** 2022-05-28

**Authors:** Autanan Wannachai, Somrawee Aramkul, Benya Suntaranont, Yuthapong Somchit, Paskorn Champrasert

**Affiliations:** 1 Optimization Theory and Applications for Engineering SYStems Research Group, Faculty of Engineering, Chiang Mai University, Chiang Mai 50200, Thailand; autanan_w@cmu.ac.th (A.W.); yuthapong@eng.cmu.ac.th (Y.S.); 2 Department of Computer, Faculty of Science and Technology, Chiang Mai Rajabhat University, Chiang Mai 50300, Thailand; somrawee@cmru.ac.th; 3 Department of Civil Engineering, Rajamangala University of Technology Lanna, Tak 63000, Thailand; benya.eng.cmu@gmail.com

**Keywords:** telemetry station, Early Warning Systems (EWS), flash flood warning, wireless sensor network

## Abstract

Floods are the most frequent type of natural disaster. Flash floods are one of the most common types of floods, caused by rapid and excessive rainfall. Normally, when a flash flood occurs, the water of the upstream river increases rapidly and flows to the downstream watersheds. The overflow of water increasingly submerges villages in the drainage basins. Flash flood early warning systems are required to mitigate losses. Water level monitoring stations can be installed at upstream river areas. However, telemetry stations face several challenges because the upstream river areas are far away and lack of public utilities (e.g., electric power and telephone lines). This research proposes hybrid effortless resilient operation stations, named HERO stations, in the flash flood early warning system. The HERO station was designed and developed with a modular design concept to be effortlessly customized and maintained. The HERO station adapts its working operation against the environmental changes to maintain a long working period with high data sensing accuracy. Moreover, the HERO station can switch its communication mode between the centralized and decentralized communication modes to increase availability. The network of the HERO stations has already been deployed in the northern part of Thailand. It results in improvements of the telemetry station’s availability. The HERO stations can adapt to environmental changes. The flash flood early warning messages can be disseminated to the villagers to increase the flood preparation time and to reduce flash flood damage.

## 1. Introduction

A flash flood is a major natural disaster that creates significant loss [1,2,3]. It happens during prolonged rainfall or extreme rainfall over a short-term period [4]. These affect river water level, which increases rapidly and floods the flood plain with unimaginable speed [5]. From the number of disasters between 1998 and 2017, floods happen more frequently than other disasters, accounting for 41% of all disasters. The number of people affected by floods was about 2 billion, with floods killing 142,088 people and causing economic loss amounting to USD 656 billion over the past 19 years [6].

A flash flood early warning system is designed to mitigate the loss. The early warning system’s goal is to give enough time for villagers in the risk area to save themselves before the flash flood impact, which is known as ‘golden time’ or ‘preparing time’ [7]. The flash flood early warning system and the flood risk management concepts can save lives and reduce losses. The flood risk area can be identified to decide appropriate action towards disaster preparedness, rescue, and recovery. Therefore, upstream river water levels should be regularly monitored to investigate the situation before the massive water flow to the downstream flood plain. A telemetry station can be installed at the upstream river to monitor water level [8]. The water level data are then transmitted to the central server for data collection and analysis. In the case of flooding incidents, warning messages will be disseminated to the villagers to prepare for the flooding situation [9].

However, the traditional flash flood early warning system faces several challenges. Accurate flood situation estimates are the key to the flash flood early warning system [10]. Flash flood prediction requires abundant water level and rainfall data. Superficially, an electronic telemetry station can be used as an automatic real-time observer [11,12]. The traditional telemetry station requires electric power and telephone line for internet network connection, troublesome to access in upstream rural areas. Solar panels and battery systems are required to be installed with the telemetry station [13,14,15,16].

Furthermore, the telemetry station should work for a long time without an electric power line connection. Solar panels and batteries are commonly used in telemetry stations. The data transmission frequency is then adjusted to be suitable to maintain the flooding incident prediction accuracy with low power energy consumption [17,18,19]. However, during prolonged rainfall, the battery energy is drained without recharging, while a high frequency of data transmission is required. The telemetry station should then adapt to the environmental changes to extend its working period and maintain highly accurate data sensing.

In addition, the early warning chain must be complete to guarantee that the warning messages can reach the villagers in the risk areas. On the technical side, the early warning chain often experiences power outages and interruptions to the telecommunication systems. During a natural disaster, the communications networks might be damaged [7]. Although the telemetry stations continue to monitor the water level, they cannot reach the central server to transmit the data due to internet outages. On the other side, the villagers cannot access the internet to get the central server’s warning messages, and as a result they cannot receive the flood incident warning. The telemetry station should therefore be able to use various communication modes to disseminate the warning message.

This research proposes hybrid effortless resilient operation stations, named HERO stations, in the flash flood early warning system. The HERO station was designed and developed with a modular design concept to be effortlessly customized and maintained. It adapts its working operation against the environmental changes to maintain a long working period with high accuracy in data sensing. Moreover, the HERO station can switch between centralized and decentralized communication modes. In the centralized communication mode, the HERO stations transmit the sensing data to the database in the central server. The water level data are represented on a website. The village leaders can decide on flooding incident preparation from the information on the website. In the decentralized communication mode, the HERO station can directly communicate with other HERO stations to examine the flash flood situation without the centralized server using short message services (SMS). In addition, the flash flood warning messages are also disseminated to village leaders directly using SMS text messaging to sustain the early warning chain. This network of HERO stations has already been deployed in the northern part of Thailand.

Compared to the related works in the telemetry station design, the telemetry stations [20,21,22] have been designed and deployed to monitor the changes in the amount of water in the natural resources. The water level data are measured and transmitted to the server via the GSM module on the mobile internet network. These techniques are similar to the proposed HERO telemetry stations. However, the other telemetry stations do not focus on their resilience in flash flood early warning systems. They cannot change the telemetry station’s communication mode on the Internet communication failures.

In contrast, the HERO telemetry stations are practically used in rural flood risk areas where the GSM communication is unstable and there is no Wi-Fi coverage. The HERO station design also focuses on the practical maintenance of the villagers. The modular design concept is also applied in HERO station architecture.

The contribution of this research is the modular design of the telemetry station, the adaptive operation mode algorithm, and the design of the decentralized and centralized communication mode switching. This network of HERO stations has already been deployed in the northern part of Thailand. The website for flash flood early warning system (https://www.pyflood.com, accessed on 11 April 2022) has already applied for flood risk areas. Moreover, the HERO station with an adaptive operation mode algorithm for switching operation modes and communication modes can be applied to disaster early warning systems such as tsunamis, volcanic eruptions, and wildfires. The modular design concept can also be applied to the sensor node development.

This paper is organized as follows. The flash flood early warning system and the hydrological principle are described in Section 2. Section 3 introduces the proposed HERO station design. The simulation configurations and results are shown in Section 4. Finally, Section 5 presents the conclusion of this paper.

## 2. The Hydrological Technique in Flash Flood Early Warning Systems

This section describes the basics of hydrological technique and the design of the traditional flash flood early warning system.

### 2.1. Hydrological Technique

In the flash flood early warning system design, the river structure and water behavior have to be well studied to analyze flooding incidents. The hydrological principle is used to describe the movement, distribution, and management of the river water. The behavior of the water and water discharge is examined for flood warning analysis. The hydraulics equation, as shown in (1), describes the flow rate (i.e., water discharge) calculation. The water discharge is calculated as multiplication of the water flow velocity and the river’s cross-section area.

(1) Q=AV

**Figure 1:**
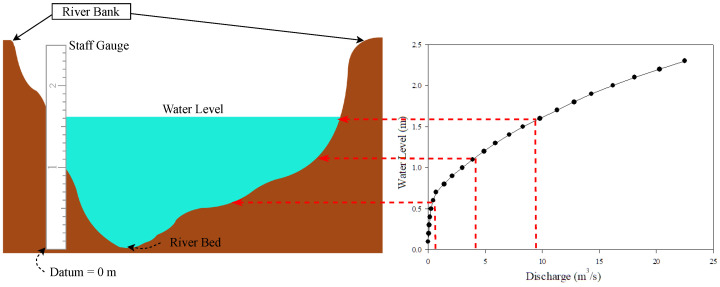
The River Cross-Section and Rating Curve.

In (1), *Q* represents water discharge (in m${}^{3}/$s), A is the river cross-section area (in m${}^{2}$), and *V* is the velocity of the water flow (in m/s). Practically, the water discharges have been collected from field surveys. The surveyors examine the river’s behavior by measuring the water discharge related to each stage of the river water level. The surveying process is time-consuming to collect most of the river water level stages, requiring about one year to manage the river’s low-flow, high-flow, and normal-flow. Then, the hydrograph, rating curve, can be plotted using a water level-discharge relationship. As shown in Figure 1, the hydrograph shows the associations of water discharge and water levels. Therefore, the water discharge can be derived from the water level.

(2) $$Q=\alpha {\left(h-h_{0}\right)}^{\beta }$$

Equation (2) demonstrates the water level-discharge relation characteristic. *Q* represents the water discharge (i.e., flow rate). $h_{0}$ is the zero gauge (i.e., datum point) level at the installed telemetry station location, and *h* is the water level measured by the telemetry station. α and β coefficients are the constant values applied to calibrate for the river’s cross-section.

### 2.2. Flash Flood Early Warning Systems

**Figure 2:**
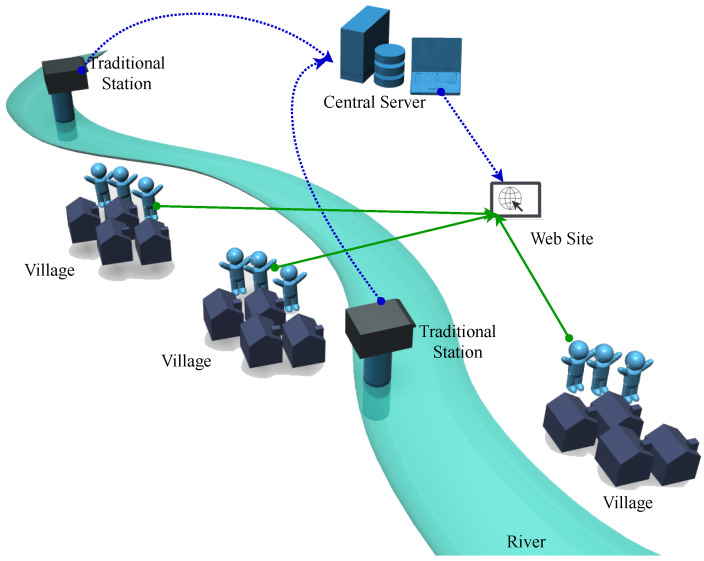
The Traditional Flash Flood Early Warning Systems.

   Generally, the flash flood early warning system consists of two main parts, which are (1) the telemetry stations and (2) the central server, as displayed in Figure 2. The telemetry station’s primary function is to monitor the water level consistently. The measured water level data are transmitted to the central server through an internet network via wired (i.e., telephone line) or wireless (i.e., mobile internet network) communication [21,23,24,25,26]. When the data arrive at the central server, they are stored in the database as the water level’s raw data. The server analyzes the data and calculates the water discharge based on the water level-discharge regression equation. Then, the server generates the flood warning messages and disseminates them to the leaders of villagers [27]. Furthermore, the flood warning message and water data are also displayed on the website as the decision-support information for the villagers in the at-risk area.

**Figure 3:**
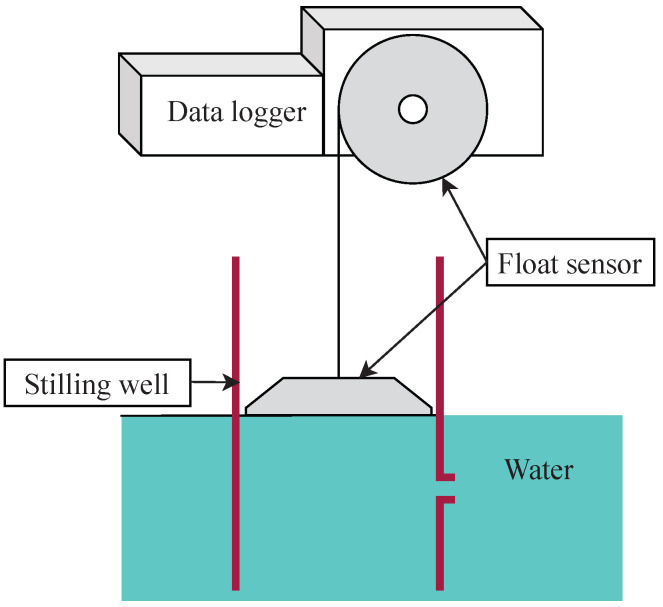
The Electronic Water Level Monitoring Station using Float Sensor.

In traditional telemetry station design, the water levels are measured using the float level gauge, as shown in Figure 3. The float level gauge consists of (1) a stilling well pipe that prevents water ripples, (2) an electronic float sensor used to measure the water level in the tube, and (3) a data logger to collect the measured water level data. The water level measurement works by a floating plate or ball moving vertically according to the river’s water level and rotating the float wheel. The float indicator interprets the float wheel rotation to the water level value. The main issue with the float system is the maintenance problems. Sediment and obstructions that come with water frequently cause the system to work improperly. The water level is measured incorrectly when obstructions block the floating plate from moving down. Solving this problem requires human labor to haul the obstacles from the tube. In Thailand, the traditional stations use the data logger to collect data and send the data packet via the telephone line. Mostly, the traditional telemetry stations are installed at the main rivers where there are power electric line providing. Thus, there is no telemetry station on the river branches and on the upstream river regions.

The new model of telemetry stations are proposed in this paper. They have been designed and invented to decrease maintenance requirements. Water levels are measured using an ultrasonic sensor to avoid contact with the water surface. Nevertheless, there are still issues since a telemetry station should be installed in the upstream river area (for early warning flash flood) where there is no electrical power. A solar panel and a battery system are required as the primary energy sources. However, the solar panel system cannot generate adequate power for the telemetry station in the rainy season. Moreover, the internet signal is unstable when the telemetry station is installed far from the city.

In this paper, the telemetry station was designed with a modular design concept to enhance effortless maintenance and improve the working time of the telemetry stations. A self-adaptive operation mode is proposed to adopt the telemetry station’s battery against the environmental changes while keeping high sensing data accuracy. Moreover, a hybrid communication mode is also proposed to keep the early warning chain during internet connection disruptions using SMS to communicate between the telemetry stations to validate the flash flood situation and to disseminate warning messages to the villagers in the flood risk area.

## 3. The HERO Station

The proposed hybrid effortless resilient operation stations, called HERO stations, have been designed to work as telemetry stations in flash flood early warning systems. The telemetry stations should work for a long period of time. The availability of the telemetry station should be high, even though the telemetry station is installed in the upstream river area where there is no electrical power or telephone communication lines. The solar panel and battery component, along with wireless communication module, should be applied along with the other sensor modules. Therefore, the modular design concept is applied in the HERO station. The modules can independently be created, modified, and replaced. The modularity of the HERO station brings effortless maintenance to the flash flood early warning system and rural villagers can diagnose and repair the station with minimum effort. The modular architecture of the HERO station is described in Section 3.1. The hardware architecture and practical installation of the HERO station are shown. Section 3.2 describes the operation mode of the HERO station. The HERO station can adapt its operation between active mode and sleep mode to deal with environmental changes. The HERO station should be resilient to examine the flash flood incident when the water level rapidly increases as well as maintaining highly accurate data sensing. Section 3.3 describes the communication mode switching mechanism in the HERO station. The HERO station sustains the early warning chain by switching its communication mode between centralized and decentralized communication modes. During flash flood events, warning messages can be disseminated to village leaders directly using SMS text messaging.

### 3.1. Modular Design

As mentioned above, the telemetry stations are responsible for being the flash flood early warning system’s observers. They have been installed at the rural upstream river, where there is no electricity or telephone communication lines. Their energy source is a battery charged by a solar panel. Practically, there are problems with a lack of energy and hardware component failure issues. These issues result in the telemetry stations not operating well. The telemetry stations require maintenance and repair periodically. However, they are installed in remote areas, and villagers with technical expertise are lacking. The maintenance and repair costs for rural telemetry stations are prohibitive. Thus, the modularity concept is designed for minimum maintenance effort [28,29,30]. The HERO station hardware components are divided into small modules that can be independently created, modified, and replaced. There is no need for high customization by adding all the modules to the HERO station. Moreover, each module is simple to diagnose using its LED status indicator. The advantages of the modular design approach are flexibility, manageability, and cost reduction. The modular design concept of HERO station can be explained in 3 sections, which include (1) hardware architecture, (2) installation, and (3) maintenance process.

#### 3.1.1. Hardware Architecture

**Figure 4:**
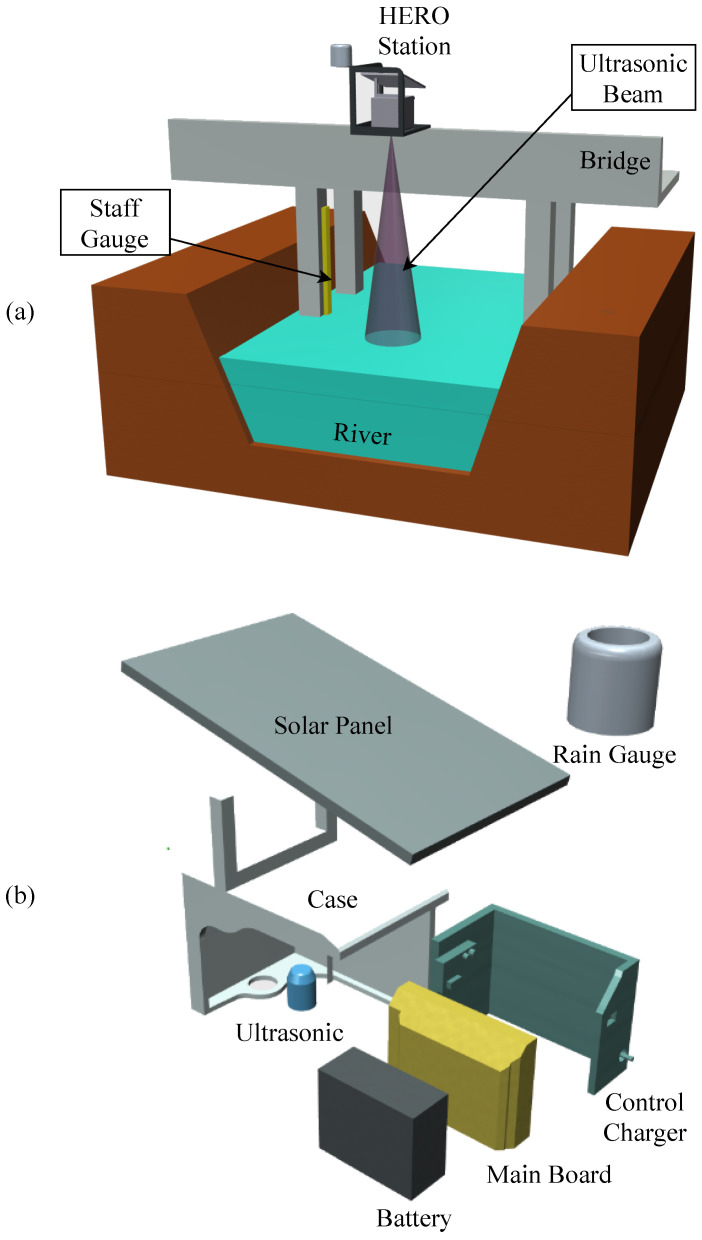
The HERO Station Design. (**a**) Station Installation, (**b**) Components of The Station.

The HERO station consists of six essential components: (1) a solar panel and a control charger, (2) electronic circuit boards (main board), (3) a battery, (4) sensor devices (e.g., ultrasonic sensor for water level measurement, rain gauge, temperature and humidity sensors for weather data measurement), (5) water proof container (case), and (6) housing connected to the bridge, as shown in Figure 4. With the modularity design concept, each component can be independently customized to fit the location of installation. The hardware architecture of the HERO station can be explained in four sections, which include (1) modular architecture, (2) sensor devices, (3) energy consumption, and (4) wireless communication.

##### Modular Architecture

**Figure 5:**
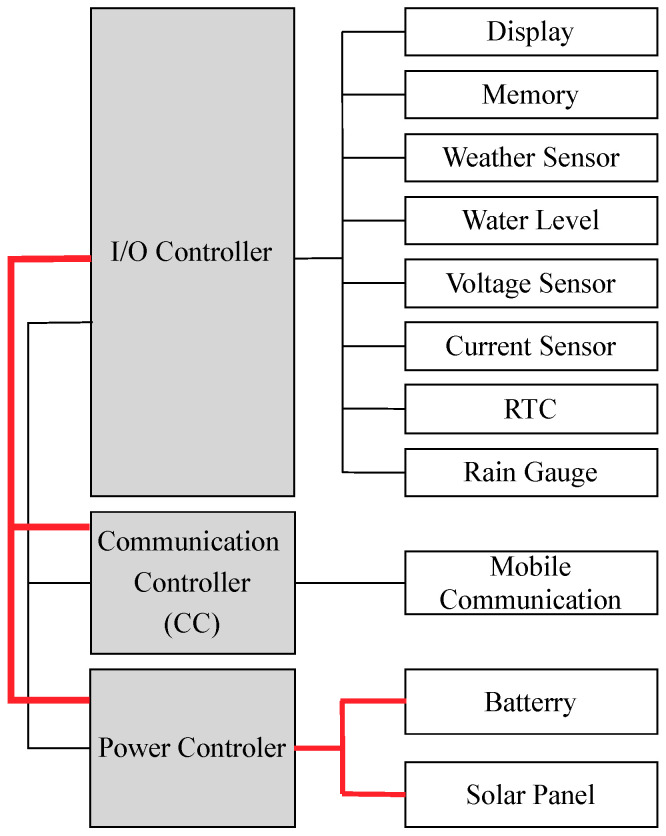
The Modular Connection Design.

The electronic circuit boards have been designed following the modularity concept. There are eleven small electronic circuit modules: (1) input/output controller (I/O controller), (2) communication controller, (3) electric power controller, (4) water level sensor (i.e., ultrasonic sensor), (5) voltage/current sensor, (6) weather sensor, (7) RTC (Real-time Clock), 8) rain gauge, (9) mobile communication modules, (10) memory, and (11) display, as shown in Figure 5. Each module is responsible for a specific function. The I/O controller module uses the I2C bus [31] as an internal communication to the display and sensor modules (i.e., weather, water level, and RTC). Each module, connected via the I2C bus, has its address for communication reference. The module interconnection protocols, and their connection addresses, are shown in Table 1.

The HERO station requires minimum maintenance effort. Each sensor module can be added to or removed from the station without reprogramming or reconfiguration. For example, when the weather sensor module is malfunctioning, it cannot send data to the I/O controller. The I/O controller then skips data processing from the malfunctioning sensor. The modular design makes the HERO station resilient to its minor failures. This procedure can be explained as follows:

- INITIAL: When the HERO station starts, the I/O controller performs sensor connection checking by looking at its module interconnection table (Table 1) and sending a sensor connection request message to each module in a round-robin manner. If the module returns an acknowledgment signal within a time limit, the module is added to the I/O controller module connection list.
- OPERATION: The I/O controller performs sensor operation readings periodically, and the frequency of this can be adjusted. The data from each sensor module is collected in the I/O controller, which reads the sensor data of the module registered in its module connection list. A time division is applied to each module communication. If the module cannot respond within the operational time limit, it is then deleted from the I/O controller’s module connection list, with the I/O controller assuming that the module is broken. Thus, the I/O controller will not communicate with that module again in the next operation.
- UPDATE: The I/O controller updates its module connection list systematically. The I/O performs initial step to recheck the module connection. In terms of maintenance, if a new module is connected to the system, the HERO station automatically registers the new module.

##### Sensor Devices

The HERO station consists of four sensor devices for measuring the water level and other environmental data. This section shows the part list of the sensor devices.

- Water Level Sensor: The maxbotic MB7066 ultrasonic sensor is used for measuring the distance between the telemetry station and the water surface. The maximum range of the sensor is 10 m. The sampling frequency is 10 Hz. The ultrasonic sensor sends the data to the I/O controller via an analog port.
- Rain Gauge: The David rain collection 6463 rain gauge sensor is used for measuring rainfall. During operation, the device can automatically drain water out. The maximum rainfall measured is 100 mm/h. The rain gauge sensor sends an interrupt signal to the I/O Controller via a digital port.
- Weather Sensor: The BME280 weather sensor is used for measuring temperature, humidity, and air pressure. The measured data are sent to the I/O Controller via an I2C bus.
- Station Energy Status: The IC MAX 471 is used for measuring the voltage of the HERO stations battery. The power consumption of the HERO station circuit is also measured.

##### Energy Consumption

The critical issue with the HERO stations for flash flood early warning systems is energy management. Because the HERO stations installation location has no electrical power line, the HERO station is designed to use its charged battery as its primary energy. To increase the lifespan, the HERO station battery is charged using a solar panel via a control charger circuit. Figure 4b shows the components of the HERO stations. The battery’s energy is harvested entirely during the daytime, while at night, on rainy days, or on days with no sunlight, the battery’s energy is consumed without charging. The total energy consumption per operation cycle ($E_{T}$) can be calculated as the summary of the power used for data transmission ($P_{t}$), monitoring data ($P_{m})$, and during the sleep period ($P_{s}$) times the times of operation (i.e., $T_{t}$, $T_{m}$, and $T_{s}$, respectively.). Equation (3) shows the calculation of the total energy consumption.

(3) $$E_{T}=\left(T_{t}P_{t}+T_{m}P_{m}+T_{s}P_{s}\right)$$

**Figure 6:**
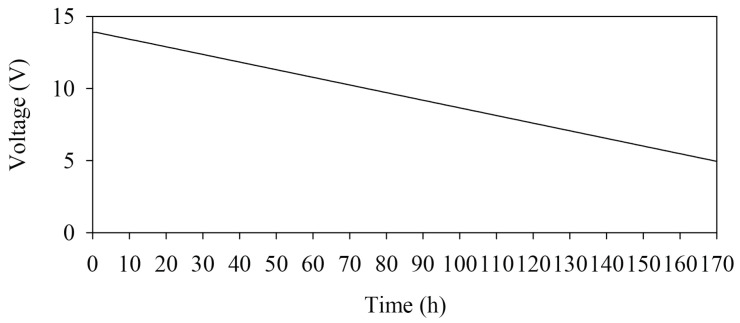
Battery Usage Testing.

The specification of the battery used for the HERO station is 12 V. DC, 21 AH, sealed lead-acid battery. The battery does not require distilled water, so it is maintenance free. It also supports high recharging numbers. It can be used for up to five years in the right environment [32]. A 40-W. solar panel is used for energy harvesting. During operation, the I/O controller requires 5 V. The DC step down module is used to convert voltage from 12 V from the battery down to 5 V. If the battery has low voltage, the DC step down module can stabilize the voltage. Figure 6 shows the experimental results of the HERO stations battery usage without energy harvesting. The X-axis shows the time in hours, and the Y-axis shows the amount of energy remaining in volts. The graph shows a continuous energy decrease. When the battery voltage is less than five volts, the HERO station’s cannot operate. From the resulting graph, the battery can be used for 170 h, or approximately seven days, on a fully charged battery.

##### Wireless Communication

The telemetry stations are usually installed at the upstream river, with no communication line for data transmission to the central server. The HERO station transmits the data through its wireless cellular communication module. The wireless communication module uses a UC20-G chip, which supports radio wave frequency at 800/850/900/1900/2100 MHz (3G UMTS) and 850/900/1800/1900 MHz (2G GSM). The UC20 chip provides a maximum data communication in HSPA+ of 14.4 Mbps for downlink and 5.57 Mbps for uplink [33]. The function of the mobile communication module, which acts as a mobile phone, is data package transmission through the internet, short message services (SMS), and phone calls.

#### 3.1.2. Installation

**Figure 7:**
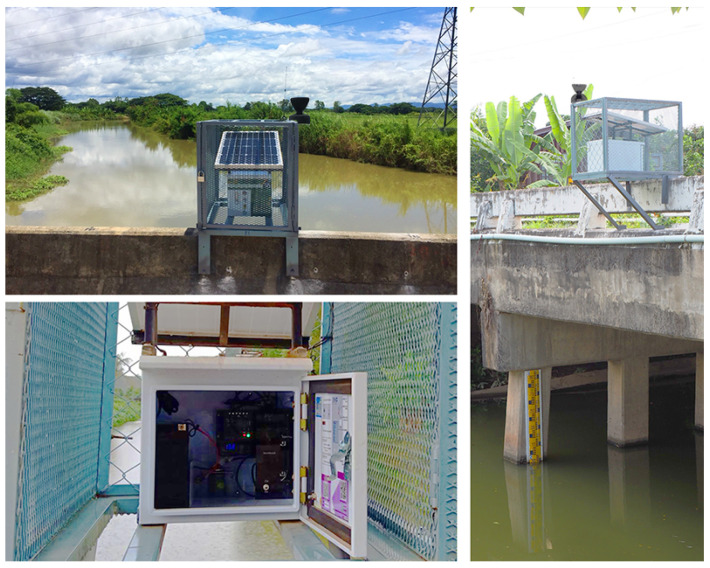
The HERO Station.

**Figure 8:**
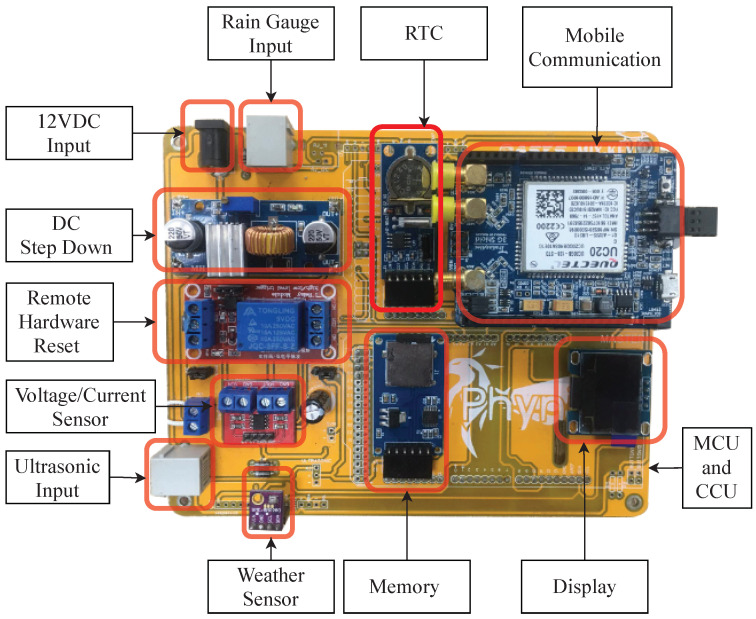
The Main Circuit Board of the HERO Station.

The HERO stations are installed in rural areas. It is designed to be portable and securely installed on concrete bridge rails. The dimensions of the HERO station are 40 cm × 30 cm × 40 cm, excluding the solar panel. The solar panel is on the top of the HERO station to reduce the temperature of the component box during the daytime. This expands the lifetime of the electronic components. The HERO station, including the solar panel, is placed in a guarding cage for physical security reasons. The rain gauge container is located on the outside of the guarding cage to measure rain levels effectively. The HERO station uses an ultrasonic sensor to measure the water level. The HERO station hangs over the bridge rail, where there is no obstruction beneath the ultrasonic sensor. Figure 7 shows the HERO stations, which is installed in a research field area. Inside the HERO station, the control unit, the mobile communication module, the display, and the sensors are built as plug-in boards on the main circuit board. The size of the main circuit board is 13 × 16 cm, as shown in Figure 8.

**Figure 9:**
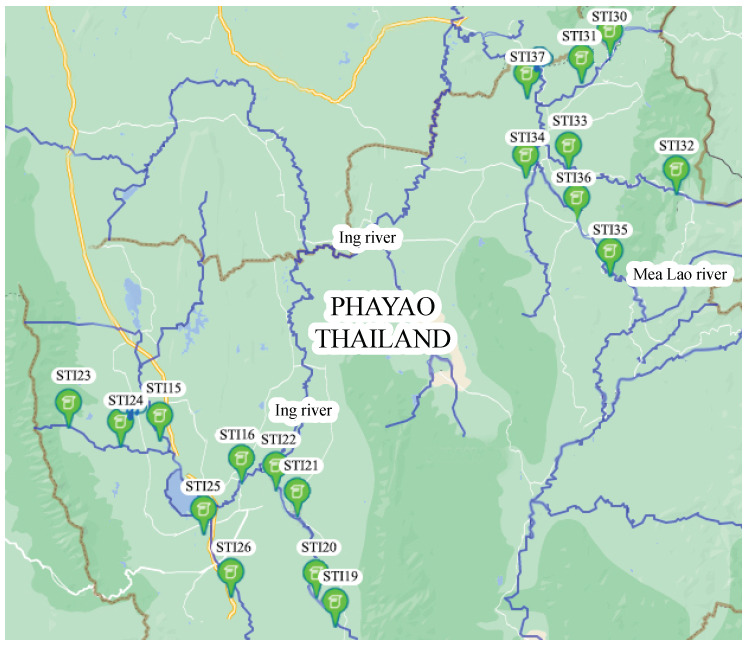
The HERO Stations at Phayao Province.

There are 18 HERO stations installed in Phayao province, Thailand. The areas in which the HERO stations have been installed are flash flood risk areas. This research focuses on two HERO stations located along a river. Figure 9 shows the locations of the installed HERO stations on the geographic map. There are at least three HERO stations installed for each stream in order to monitor water levels and warn of flash floods. The two HERO stations are installed at the upstream and midstream. The rest are installed on the river before the water flow to the village.

**Figure 10:**
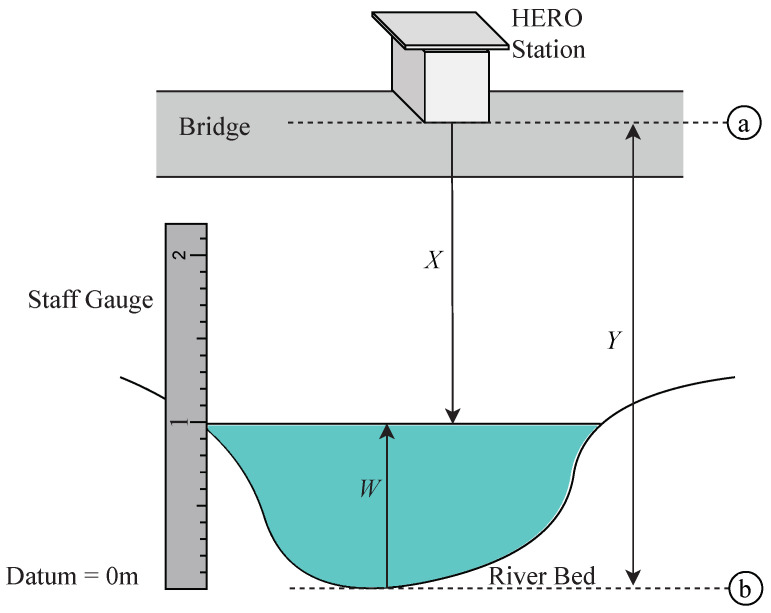
Water Level Measurement.

To measure the water level without contacting the water’s surface, the HERO station uses an ultrasonic sensor device, which emits a sound that humans cannot hear. It will also determine the time when the sound reflection returns and calculate the distance between the ultrasonic sensor and the water’s surface. Figure 10 demonstrates the model for calculating the water level from a distance measured by the ultrasonic sensor.

(4) W=Y−X

Equation (4) explains the water level (*W*) calculation. *Y* is the distance from the HERO station base to the zero points of the surveyed river cross-section which are (a) and (b) in Figure 10, respectively. The distance is explored from the field survey. *X* is the distance measured by the ultrasonic sensor. For calibrating, the calculated distances are compared with the staff gauge, which is installed beside the bridge to measure the water level. The photo image of the installed staff gauge is also shown in Figure 7.

However, the ultrasonic sensor data may come with noise due to the actual floating objects in the river. Therefore, the HERO station filters the noise of sensing data using a moving average filter. The moving average is a simple low pass filter process [34,35,36]. The moving average filter is implemented on the HERO station to decrease noise and consume small computing resources (i.e., CPU and memory). The HERO station performs a data sensing process every second. Then, the HERO station performs the moving average process every two minutes and transmits the data to the central server. Compared with the other filtering methods (e.g., Kalman filter [37], Adaptive k filter [38], Fuzzy filter [39], and ARIMA [40]), these filtering methods may return better noise-canceling data. However, these filtering methods are more complex and require more computing resources which are challenging to work in the microcontroller unit of the station.

#### 3.1.3. Maintenance

In the case of a hardware component failure, the HERO station can continually operate. For example, if the RTC is broken, the HERO station can continue to send data packets to the central server. In this case, the data will be timestamped using the server time instead of the RTC. Moreover, if any sensor module is broken, the unmeasured data are not included within the data packet transmitted to the central server. Therefore, because of the missing data, the central server will be informed that the sensor has failed. Then, the maintenance process is started by volunteer villagers living close to the station. A diagnostic fault check is performed to identify the failed module. In the HERO station’s modular design, each module has an LED status indicator. The LED status indicator lights green when the module is working properly. If there is no green LED light or the LED status indicator blinks to show an error code, the module should be repaired. The broken module can be replaced in plug-and-play manner, with the volunteer villagers unplugging and delivering the broken module to the research lab, where a working replacement module will be released. During this maintenance process, the HERO station can continue to operate with no idle time.

### 3.2. Adaptive Operation Mode

It is necessary to install the HERO station for flash flood early warning system at a point upstream, where there is no power electric transmission line connection. The solar panel and battery are vital energy sources for the HERO station operation. The HERO station can effectively manage power consumption by adjusting for suitable data transmission frequency according to the water changes. The adaptive operation modes are designed for the HERO station to perform highly accurate data sensing over a long working period.

The HERO station can adapt its operation between active and sleep mode. In the active, or transmission operation, mode, the HERO station measures and collects the sensor data from its sensors and then transmits sensor data to the central server to analyze and decide whether or not to disseminate the warning message to the villagers. While in sleep mode, the HERO station only measures and collects sensor data, but there is no data transmission to the central server in order to preserve energy consumption. To adapt operation, the HERO station analyzes the water level data to determine if it is similar to the previous data over a long-time span, which means there is no flash flood. Then, the HERO station continues its sleep mode period. However, when the water level changes dramatically, the HERO station works in active mode and continues transmitting the sensed data to the central server, which is explained in Algorithm 1. The water level threshold cannot be used as a constant because in each season the average water level is different. Therefore, data analysis is required to estimate the water level situation, which is explained in Algorithm 2. Then, the active and sleep mode adaptation is explained in Algorithm 3.
**Algorithm 1:** Main Algorithm

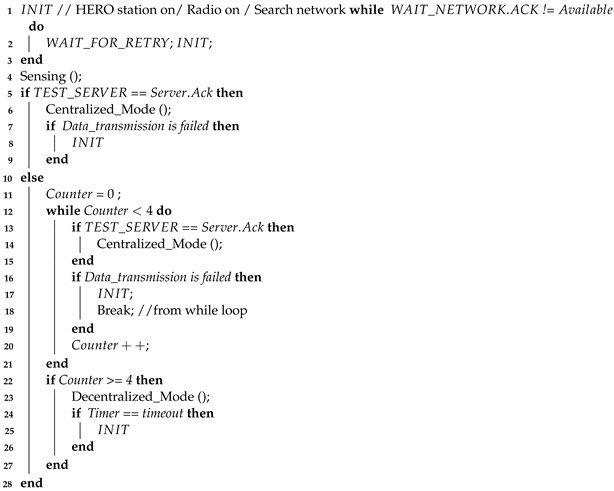


   Algorithm 1 (Main Algorithm) explains the operation of the HERO station. The process begins with the HERO station turning on *INIT* state. Then, the mobile communication radio is on and searches for the network (telephone network). If the *WAIT_NETWORK.ACK* is not *Available*, the HERO station waits to retry and returns to *INIT* state again. The HERO station searches for the network until a response is given. When the *WAIT_NETWORK.ACK* is *Available*, the HERO station calls *Sensing ()* procedure and sends a data packet to test the central server in the *TEST_SERVER* state. If the HERO station receives a response from the central server (*Server.Ack*), the HERO station enters *CENTRALIZED_MODE* state. If the data transmission fails but the central server returns *Server.Ack* in time, the HERO station will return to the *INIT* state again. On the other hand, if the HERO station does not receive a response from the central server three times, the HERO station will enter into *DECENTRALIZED_MODE* state to prevent the HERO station from using too much power to transmit data while unable to contact the central server. To switch communication mode back to the centralized mode, the HERO station uses a timer (timer=timeout) to switch to *INIT* state periodically.
**Algorithm 2:** Flood Starting Situation Classification

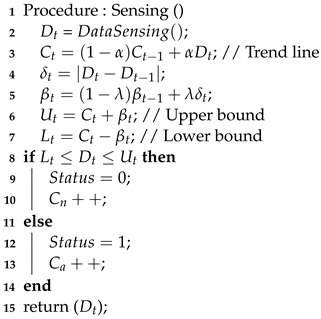


**Algorithm 3:** Sleep and Active Mode Adaptation


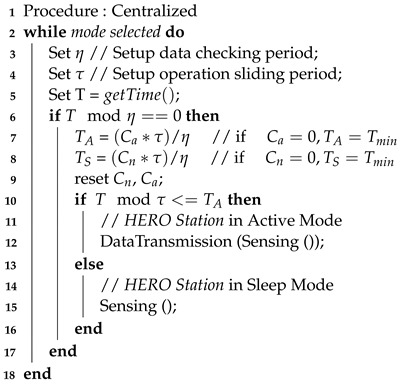



Algorithm 2 demonstrates how to classify the start of flood situation using sensed water level data. The water level data are used to classify this situation using boundary values, the upper bound ($U_{t}$) and the lower bound ($L_{t}$), which are calculated using the *EWMA* control chart. *EWMA* chart or exponentially weighted moving average chart is weighted the data in decreasing order. The recent data are high weighted while the most distant data contribute a less weighted. As such, the boundary values can be adjusted based on a series of previous water level data ($D_{t}$). The boundary size is also adjusted to the changes in water levels ($\delta_{t}$). In the low flow season, the water level does not change the so the boundary size becomes smaller. In the high flow rainy season, the water level changes and so the boundary size becomes bigger. If the water level data values stay within the bounds, the status of the start of a flood situation is determined as False (0). Otherwise, the status of flood starting situation is set as True (1). The counting times of the status in no flood situation and active flood situation are also recorded in the global variables $C_{n}$ and $C_{a}$, respectively. These variables are used to calculate the period of active and sleep modes in Algorithm 3. Algorithm 2 makes the HERO station learn changes in water level data.

Algorithm 3 explains the procedure for active and sleep mode adaptation. The process is used to adjust the period that the HERO station works in active mode ($T_{A}$) and in sleep mode ($T_{S}$). In the algorithm, η is the operation checking period. The constant value (τ) is the sliding windows time. $C_{n}$ and $C_{a}$ are the numbers of status counts in no flood and active flood situations, respectively. $T_{min}$ is the minimum time period. The periods of active and sleep modes are calculated using (5) and (6), respectively. These values are used to determine which operation mode will be performed. In a flooding situation, the sensed data value (i.e., water level) increases rapidly. When the sensed data ($D_{t}$) are out of boundary, the number for $C_{a}$ increases. Therefore, the HERO station operates in active mode to obtain the water level data and transmit the data to the central server in a high frequency. In the case of no flooding incident, the period in sleep mode is long in order to conserve energy. The central server assumes that the current water level data are the same as the previously collected data. This may lead to low accuracy of data, but the compromise is the energy saving of the HERO station. However, during the sleep operation mode, the HERO station continues to read the data and check the boundary. When the data are out of boundary, the HERO station wakes up from sleep mode and enters active mode. The adaptation in the HERO station’s operation mode results in extended working time span and maintains high accuracy of data in the central server. This is especially useful during the rainy season, when there is a short period of sunlight to charge the battery. The necessary outcome from the early warning system for flash floods is that the stakeholder (i.e., the village leader, local authorities, and villagers) can receive the warning messages when a flash flood occurs. During flash flood incidents, internet communication may be shut down, and the early warning chain is broken. However, mobile phone short messages (SMS) are more reliable [41]. Although the sensed data can be collected by the HERO station, they cannot be transmitted to the central server because of the internet failure. The stakeholder cannot receive the flood incident warning, which can result in significant losses. The adaptable operation modes work only during the centralized communication mode, which has no communication failures.

### 3.3. Hybrid Communication Mode

The HERO station is designed to perform hybrid communication modes to guarantee that the warning message will be disseminated. Under normal circumstances, the HERO stations work in the centralized communication mode. They periodically sense the data and transmit it to the central server to analyze the flood situation. The flood incident warning message is disseminated through the website and social media (i.e., Facebook messenger and Line applications). Whenever a flood occurs at the same time as a communication problem, the HERO station can switch to the decentralized communication mode. Based on the water data sensed by the HERO station and its neighbor stations, the HERO station analyzes the water situation and decides to disseminate the flood incident warning messages directly to the village leaders through the SMS network.

**Figure 11:**
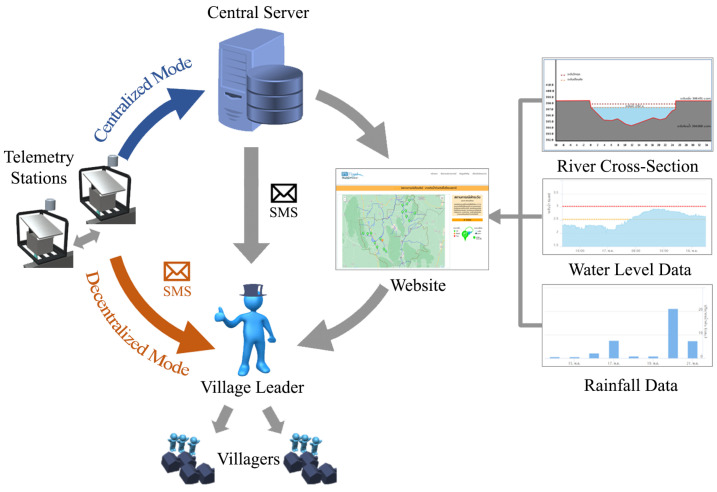
Decision Support System in Flash Flood Incident for Village Leaders.

The network of HERO stations works as the decision support system in flash flood incidents for the village leader. Village leaders require to receive spatial-temporal data on river water level and the amount of water falling in the rain within a given time and area. Working as the decision support system as shown in Figure 11, village leaders receive incident information in three ways which are (1) information on the website, (2) warning messages (e.g., SMS, Line Application messages, and social media) from a central server when the water level is over the threshold, and (3) direct warning SMS from the HERO stations in decentralized communication mode when a flood incident is detected but no network communication. In centralized communication mode, the website shows the flood information in real-time. The website shows the information of the river water level and rainfall on several options (e.g., maps, graphs, river cross-section graphics, and text summary of flood incidents with the color to represent critical flood levels. Figure 11 shows the visualization of the website. First, the river cross-section graphic is plotted based on the survey data, and the water level changes in real-time. Secondly, the graph represents the water levels and the time that the water level increases beyond the warning threshold. Moreover, the water level graphs between two HERO stations can estimate water travel time. Finally, the bar graph represents the rainfall data. Therefore, the village leaders can decide to warn the villager in the area before the flood comes.

**Figure 12:**
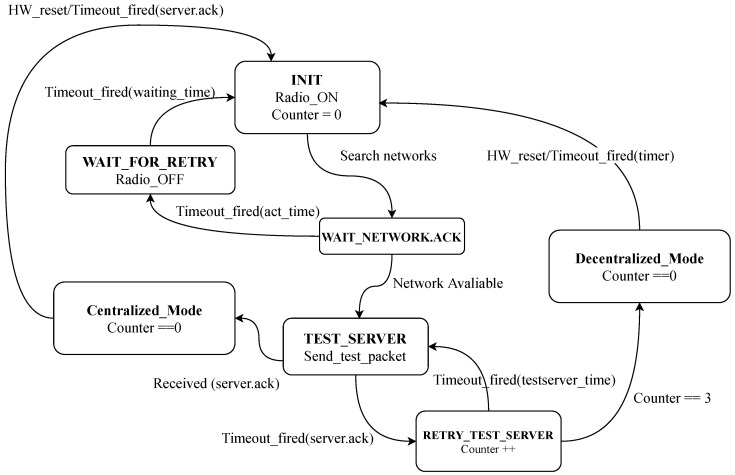
FSM of The Hybrid Communication Mode.

Figure 12 explains the finite state machine (*FSM*) of the HERO station’s communication mode switching. The operation begins with the initial state (*INIT*) in which the station starts the mobile communication module service to wait for the network connection. Then, the state turns to *WAIT NETWORK.ACK*, meaning the station waits for the acknowledgement message to return from the mobile communication gateway. If the acknowledgement message does not return within the prescribed time (*ack time*), the mobile communication module service will be stopped and the status will change to *WAIT FOR RETRY* state. The HERO station then has to wait for *wait time* seconds and returns to the initial state again. However, if the HERO station receives the acknowledgement message back from the communication network gateway, meaning the HERO station is connected to the network, the state changes to *TEST SERVER* state. During this state, the HERO station sends the test packet to the central server to check the connection. If no acknowledgement message returns within the specified time (*test server time*), the station will turn itself state back to *TEST SERVER* state and re-sends the test packet to the central server. This server connection test will be tried three times (Counter==3). If the station cannot communicate with the central server, it switches the communication mode to decentralized (*DECENTRALIZED MODE*). Otherwise, the station performs in a centralized communication mode, and the counter parameter will be reset to zero. Moreover, the HERO station will automatically reset (*HW reset*) twice a day to maintain stability. This procedure performs the HERO station’s state return to the initial state.

(5) $$T_{A}=\left\{\begin{array}{cc} & \frac{C_{a}*\tau }{\eta },\;ifC_{a}>0\\ & T_{min},\;otherwise \end{array}\right.$$

(6) $$T_{S}=\left\{\begin{array}{cc} & \frac{C_{n}*\tau }{\eta },\;ifC_{n}>0\\ & T_{min},\;otherwise \end{array}\right.$$

#### 3.3.1. Centralization

**Figure 13:**
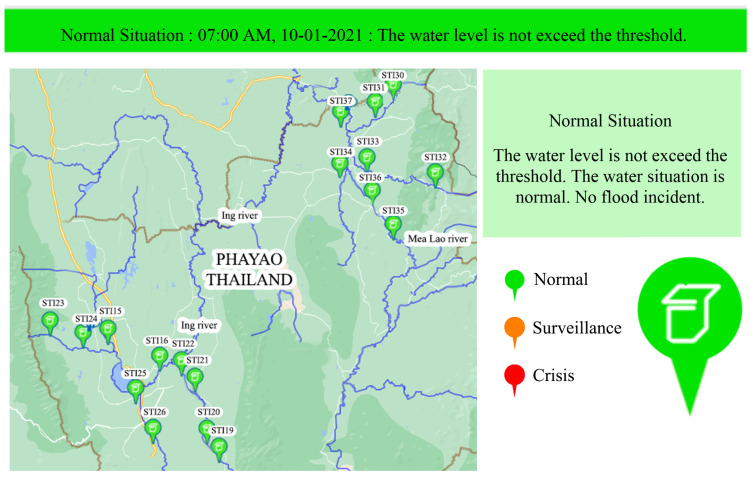
The Flash Flood Early Warning Website.

The HERO station commonly works in the centralized communication mode with a good internet connection. The sensed data will be collected and transmitted to the central server through a mobile data network. The data packet details are shown in Table 2. The sensed data consist of data, time, station ID, distance between the HERO station and water surface, rainfall, temperature, humidity, air pressure, battery voltage, battery current, and packet status, which identifies the water situation. When the data packet arrives at the central server, the data are processed and stored in the database. The flash flood early warning website was designed to disseminate the flooding incident and other related information, as shown in Figure 13. The water situation is displayed as a colored pinpoint on the geographic map. There are three colors (Green, Orange and Red) to represent the water situation. Table 3 provides the definitions of the colors representing the water situation and the warning text that is disseminated on the website. The HERO station has its threshold to determine the water situation. The experienced villagers in the local area set the threshold. In addition, real-time and historical water level data are presented on the website for use as information to support water resource management. Once the flooding incident occurs, the warning message are also directly sent to the stakeholders through Facebook messenger and Line application to manage the flood incident.

#### 3.3.2. Decentralization

During communication failures, when the HERO station cannot contact the central server and OR the stakeholders cannot access the early warning website, the HERO station will switch to operate in the decentralized communication mode. Algorithm 4 demonstrates how the HERO station works in this mode. The sensed data are collected and their status determined, as explained in Algorithm 2. If the data status is equal to zero, the HERO station only stores sensed data within an external drive. Otherwise, the HERO station sends the request message to ask for the neighbor’s (NTS) status. If the HERO station receives a message from NTS, the HERO calculates voting weight ($W_{v}$) based on the neighbors’ status ($S_{d}$), the HERO station’s weight ($W_{t}$), and its status ($S_{t}$).

Equation (7) shows the calculation of the HERO station’s weight calculated based on the distance between the HERO station and its neighbor located in the same river stream, where $d_{s}$ is the distance between the HERO station and its neighbors. $d_{min}$ is the distance between the village and the village nearest to the HERO station. $d_{max}$ is the distance between the village and the village furthest from the HERO station. The number of situation rechecks (*r*, weight limit) is the value setting for the HERO station to check the situation of flooding in decentralized mode. Usually, The HERO station checks the situation of water level data every sensing data. For example, when r=2, When the water level is out of bound once, the HERO station needs to recheck the flooding situation one more time using voting weight ($W_{v}$) or warning weight ($W_{w}$).

If the voting weight is greater than the weight limit (*r*), the warning message will be directly disseminated to the village leader through the SMS network. If there is no response from any neighbor, the warning weight ($W_{w}$) is calculated by adding the HERO station’s status at time *t* ($S_{T}\left(t\right)$) with its status at time *t*+1 ($S_{T}\left(t+1\right)$). If the warning weight is greater than weight limit, the warning message will be sent to the village leader through the SMS network.
**Algorithm 4:** Decentralized Communication Mode

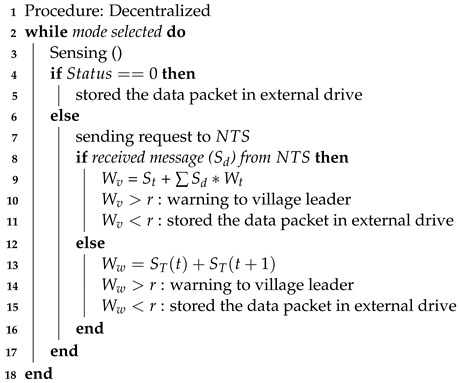


Moreover, there is a threshold value that represents the highest water level before flooding occurs. This threshold value is explored in the field survey. If the sensed water level is greater than the threshold, the HERO station also disseminates the warning message to the village leader in order to prepare for a flooding incident. Table 4 shows the structure of the data packet for warning of a flood situation. The data packet consists of a date, time, station ID, water level, and warning text message.

(7) $$W_{t}=2\frac{d_{s}-d_{min}}{d_{max}-d_{min}}$$

## 4. Simulation and Result

**Figure 14:**
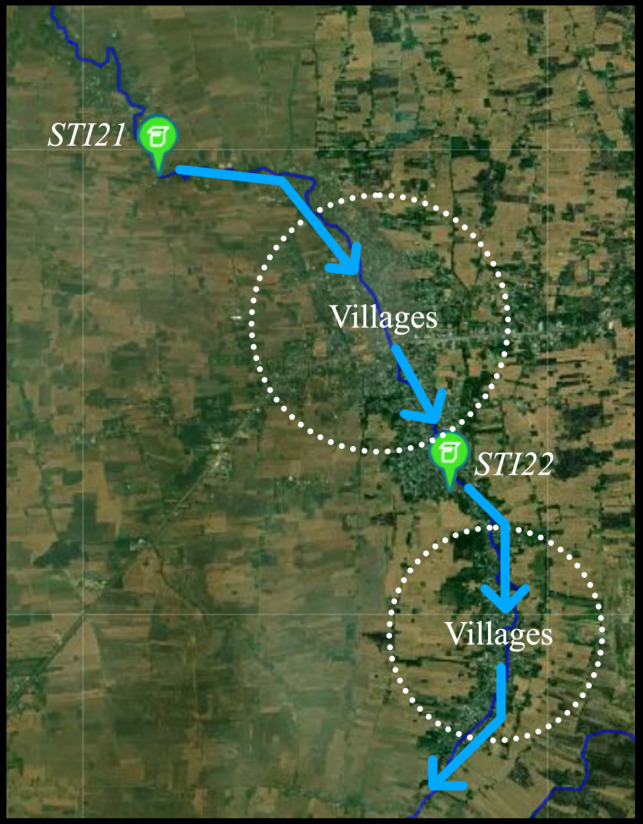
The Installation Area (*STI21*, *STI22*) and The Village Location.

This section evaluates how the HERO station automatically adjusts its operation mode according to environmental and communication conditions; moreover, the accuracy of an early warning in all the communication modes is measured. The HERO station is installed at the upstream area (as shown in Figure 14) to collect the actual data and create the rating curve to evaluate the amount of water before disseminating the warning message to the villagers. The collected water level data from the experiment is used in the simulation for mode switching according to the flood situation.

The performance matrix evaluates the HERO station in terms of adaptability to environmental changes, accuracy of flooding incident data (in centralized communication mode), dissemination of the warning message before the flood (in decentralized communication mode), parameter impact, and energy efficiency.

### 4.1. The Result of The Experiment

**Figure 15:**
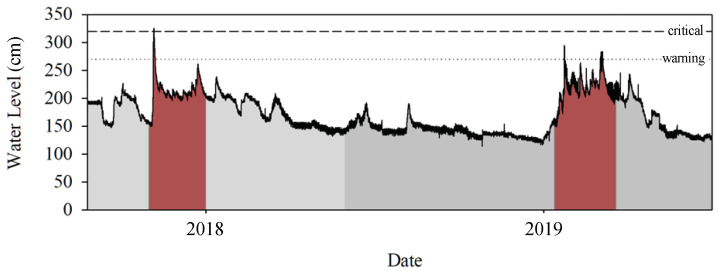
Water Level Data from HERO Station (years 2018–2019).

The HERO stations have been deployed since 2018 in Mueang District, Phayao Province, Northern Thailand, to track water levels, which are collected and transferred for storage in the database on the control server every 5 min. Figure 15 shows the graph of the collected water level data over two years. During the rainy season each year, from August to September (red highlight), a few flooding incidents occurred when the water level is higher than the warning and critical levels, which are represented by the dotted and dashed line in the graph, respectively.

**Figure 16:**
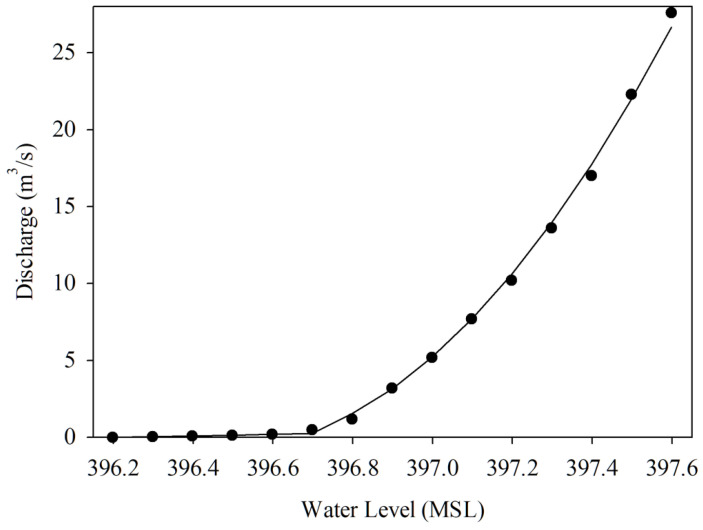
Rating Curve of The HERO Station (*STI21*).

**Figure 17:**
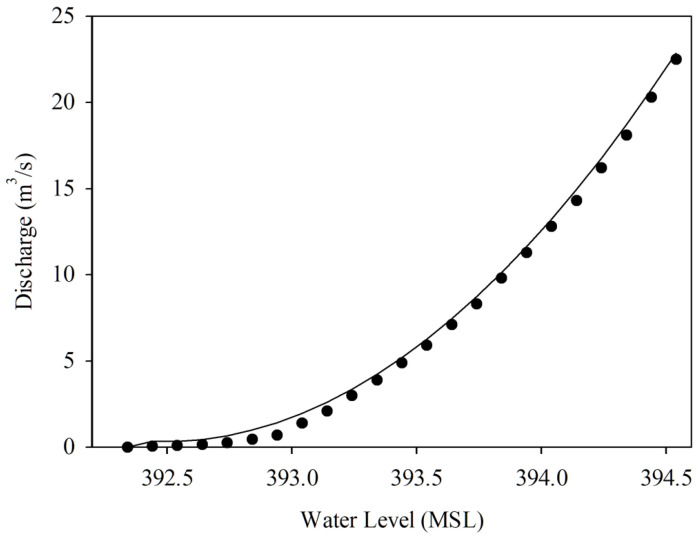
Rating Curve of The HERO Station (*STI22*).

In the experimental area, two HERO stations called *STI21* and *STI22* were installed on bridges located upstream and downstream of the same river. The water level data, together with the river velocity and a cross-section of the river collected from the field survey, was used to plot the rating curve graph of *STI21* and *STI22*, as shown in Figure 16 and Figure 17. These rating curve graphs are used to estimate the water discharge (the water volume) in the river when we know the water level. Thus, the village leader use the rating curve to determine flooding incidents.

**Figure 18:**
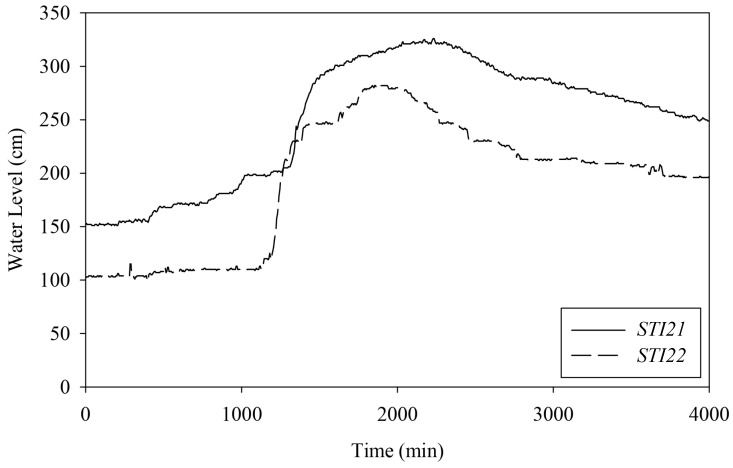
Water Level Data of *STI21* and *STI22*.

Figure 18 displays the water level data sets from *STI21* and *STI22* during the flood incident in August 2019. Local authorities have confirmed the data sets for their accuracy and will be used for simulation in this paper. The graph shows the water level data from time 0 to 4000 min (approximately three days). At the 1000th min, the water level measured by *STI21* increased dramatically. At the same time, the water level measured by *STI22* increases at the 1500th min. It means the significant water volume has occurred upstream, and this water volume will come along the river to the village located downstream within 500 min (about 8 and 1/2 h). This golden time helps the villagers prepare for the coming flood and reduce losses.

### 4.2. The Result of Simulation

The objective of the simulation is to assess the HERO station’s efficiency in terms of operation mode adaptation, data accuracy, and energy lifetime. Table 5 displays the setup parameters used in the simulation. The 2400 packets data set comes from the actual water levels during the experiment (800 packets from the centralized mode simulation, and 2400 packets from the decentralized mode simulation). (α) and (λ), the constant values used in the data sensing algorithm (Algorithm 2), are set to 0.65 and 0.1, respectively. The data checking period (η) is 250 min. These are used to determine the ratio of the operation mode switching between active and sleep. The sliding windows time (τ) is equal to 50 min. This value is the time that will be set or used to determine the operation mode switching ratio. The frequency of data sensing is every five minutes. The energy consumption for data transcription is 180 Jules per packet. The energy consumption during sleep mode is 0.72 Jules per second. The HERO station senses both energy consumptions during the experiment.

#### 4.2.1. Centralized Communication Mode

**Figure 19:**
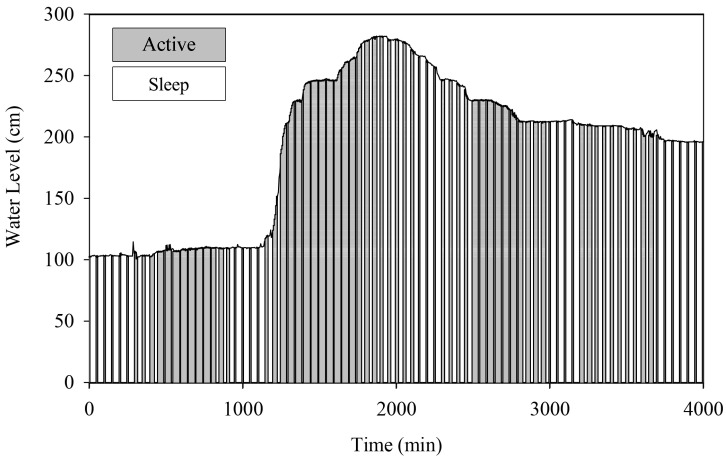
Centralized Communication Mode (*STI21*).

**Figure 20:**
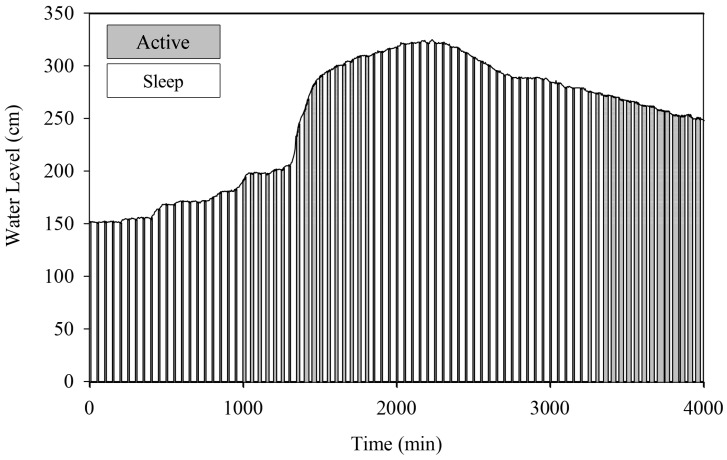
Centralized Communication Mode (*STI22*).

The HERO station switches between transmission and sleep operation modes according to the water situation in centralized communication mode. The aim is to save the lifetime of battery consumption. Figure 19 and Figure 20 reveal the proportions for active and sleep operation modes of *STI21* and *STI22*, respectively. The X-axis displays the total simulation time in minutes, and the Y-axis shows the water level. The graph shows the operation mode of each interval time using the colored band. The results show that the HERO station primarily operates in the sleep mode during normal situations. This causes the HERO station to reduce total energy consumption and expand overall battery operation lifetime. However, during heavy rain (the water level data dramatically increase over a short time), the HERO station mainly operates in active mode.

#### 4.2.2. The Accuracy of The Water Level Data in Centralized Communication Mode

**Figure 21:**
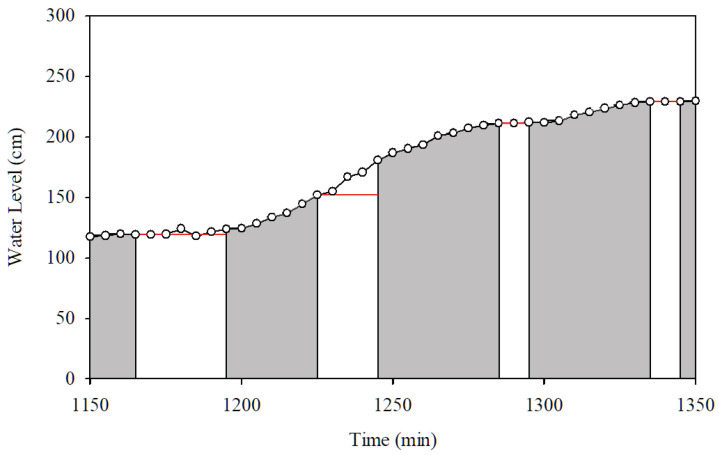
Data Accuracy of HERO on Centralized Communication Mode (*STI21*).

The accuracy of the water level data is the main issue that has to be considered during the flood incident. During active mode, the actual water level is sensed and transmitted to the central server. However, under normal conditions, the water level data are measured and stored in the HERO station. Consequently, the central server will use previous data to analyze and disseminate the water situation. Figure 21 shows the actual water level data measured by the HERO station, represented by white dots. They are compared with the water level data disseminated by the central server, represented by the red line. Means absolute error (MAE), as demonstrated in (8), is used to determine the difference in the measurement of the water levels between the actual water levels measured by the HERO station during the experiment and the water levels resulting from the simulation ($e_{t}$). The results show that the MAE is just 0.55%. The MAE points out that the accuracy of the water level within operation mode switching is acceptable.

(8) $$\mathrm{MAE}=(\frac{1}{n}\sum_{t=1}^{n}|e_{t}|)$$

#### 4.2.3. Parameter Setting Impact

The data checking period (η) specifies the number of previous data or duration used to determine the operation mode proportion between active and sleep mode for the next period. During the simulation, this parameter is set to multiple values to evaluate the efficiency of the HERO station. Table 6 presents the MAE and the average percentage of energy consumption improvement affected by the varied duration parameters. The results reveal that the more durations there are, the higher the MAE will be. However, the accuracy is still acceptable as long as the duration value is not set too high. Furthermore, the duration value has a positive effect on energy consumption. These ensure the HERO station can reliably monitor water levels when performing operation mode switching in centralized communication mode.

#### 4.2.4. Decentralized Communication Mode

**Figure 22:**
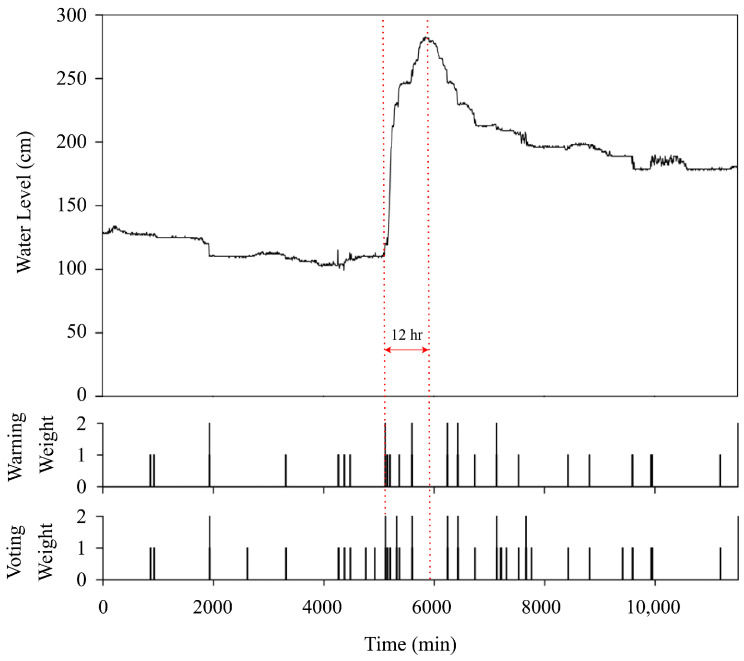
Decentralized communication Mode (*STI21*).

**Figure 23:**
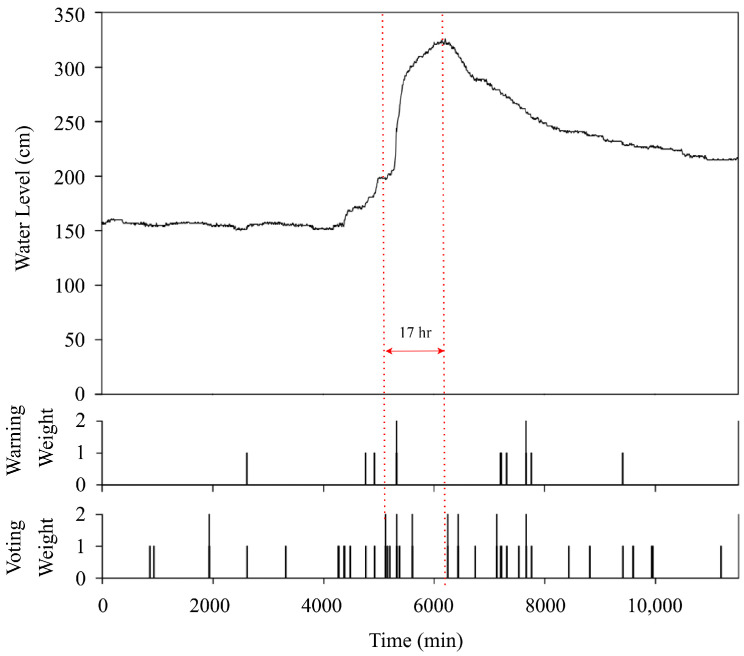
Decentralized communication Mode (*STI22*).

In the decentralized communication mode, the HERO station is not able to communicate with the central server. Thus, the HERO station must determine the water situation and disseminate the warning message. Figure 22 and Figure 23 show the operation to resolve the water situation during the decentralized communication mode of *STI21* and *STI22*, respectively. The y-axis consists of three parts: the water level relative to time, a warning weight of the HERO station, and a voting weight collected from its neighbor. The x-axis represents the time in minutes. The HERO station will determine the serious water situation when warning or voting weight exceeds the weight limits (>2 in this simulation). Then, the HERO station will disseminate an SMS about the serious water situation to alert the community leaders. As seen in Figure 23, the HERO stations sent the warning message out based on the rapid change in water levels in the 1935th, 5120th, 5325th, 5650th, 6245th, 6435th, 7135th, and 7665th min. As such, the community leaders can be informed of the water situation and make the right decision for water management.

In the centralized communication mode, the HERO station continuously transmits data to the central server. The village leaders and villagers can monitor the river water level and rainfall on the website. The flood incident warning messages are sent to the village leaders when the water level exceeds the warning threshold via the central server. The village leaders investigate the flood incident using the river water level and rainfall. Then, the village leaders can decide to warn the villagers in the flood risk area before the flood comes.

In the Internet communication failure in the flooding area, the adaptive operation mode in the HERO station switches its communication mode to a decentralized communication mode. The decentralized communication mode starts when the HERO station cannot contact the central server. If the water level is out of bounds, warning messages are sent to the village leaders via mobile network using SMS. The proposed voting with a warning weight algorithm can reduce false-positive errors for flood incident warnings.

#### 4.2.5. Energy Efficient

Energy efficiency is an essential concern of the HERO stations since they are installed in areas lacking electricity. Most energy is consumed for data transmission to maintain data accuracy. The energy consumption of the HERO station is described as Equation (9). The total energy consumption ($E_{Total}$) is a combination of the amount of power used for data transmission ($P_{t}$) multiplied by the number of packets (*N*) and the amount of power used in sleep mode ($P_{s}$) multiplied by the sleep time duration ($T_{s}$). The power used for data transmission ($P_{t}$) includes the HERO station’s sensing energy consumption, analyzes data, and sends the data packet to the central server. The power used for sleep mode ($P_{s}$) includes the HERO station’s energy consumption of sensing data, analyzes data, standby, and sends the data packet.

(9) $$E_{Total}=\left(NP_{t}+T_{s}P_{s}\right)$$

(10) $$E_{imp}=\frac{E_{T}-E_{P}}{E_{T}}\times 100$$

Equation (10) shows the calculation of the percentage of energy improvement ($E_{imp}$). It is used to assess the energy consumption improvement of the HERO station in centralized communication mode. $E_{T}$ is the total energy consumption of the traditional telemetry sr tation used to transmit data for 4000 min in simulation. $E_{P}$ is the total energy consumption of the proposed telemetry station (The HERO station). The results show that the HERO station can improve energy consumption by 38% compared with a traditional telemetry station.

**Figure 24:**
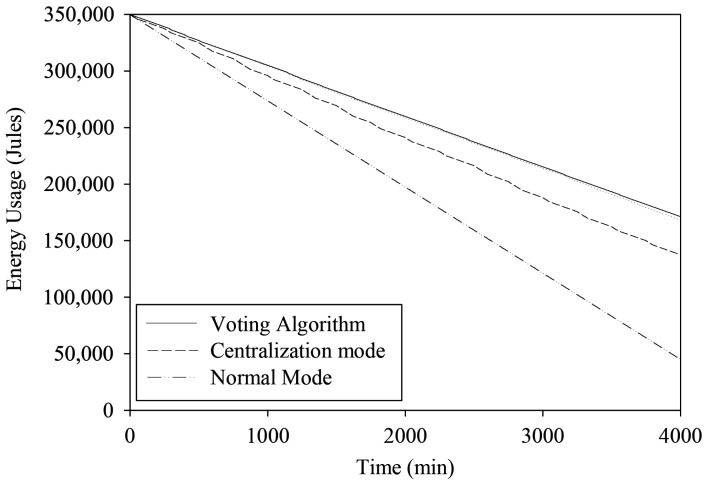
Energy Usage.

Figure 24 shows the energy usage of the HERO station (*STI21*) in the centralized communication mode compared with that in the decentralized communication mode. The graph also compares energy usage with the traditional telemetry station. The results show that the HERO station operating in the centralized and decentralized communication mode uses less energy than an traditional telemetry station, at 38% and 42%, respectively.

## 5. Conclusions

This paper proposes the HERO station, which was designed and implemented using three concept designs (1) the modular design, (2) the adaptive operation mode, and (3) the hybrid communication mode. The HERO station was designed and developed with a modular design concept to be effortlessly customized and maintained. It can reduce maintenance costs and make HERO stations highly available for flash flood EWS. The adaptive operation mode allows the HERO station to improve data accuracy while maintaining energy efficiency. The hybrid communication mode also means the HERO station can disseminate flash flood early warning messages to villagers even in Internet communication failures. The HERO station can adapt its operation and communication modes to environmental changes. The results show that even if the HERO station works mostly in sleep mode, sensing data errors from receiving the missing water level data are acceptable (MAE = 0.55%). The hybrid communication mode allows the HERO station to maintain a warning message chain even with environmental changes and network problems. The HERO station is able to automatically switch between centralized and decentralized communication modes depending on the network situation. The 18 HERO stations have been installed and used in rural flooding risk areas. The modular design has allowed the HERO station to be continuously operated by local villagers since 2018. The HERO station design concept can be applied to disaster early warning systems (e.g., tsunamis, volcanic eruptions, and wildfires). The modular design concept can also be applied to the industry sensor node development.

## Figures and Tables

**Table 1:** The Module Interconnection Table.

Module	I2C	Analog	Digital	SPI	Serial	Address/Pin
Input						
Water Level		✓				
Weather Sensor	✓					0x77
Rain Guage			✓			Interupt
Voltage Sensor		✓				
Current Sensor		✓				
RTC	✓					0xa3
Output						
Display	✓					0x3C
Memory				✓		
Mobile Communication					✓	
Power Controller			✓			
Communication Controller					✓	

**Table 2:** The Data Packet (Centralized Communication Mode).

8 bytes	4 bytes	5 bytes	4 bytes	2 bytes	2 bytes	3 bytes	3 bytes	2 bytes	2 bytes	1 bytes
Date	Time	Station ID	Ultrasonic	Rain	Temp	Humidity	Presure	Battery Voltage	Battery Current	Status

**Table 3:** Colour, Situation, and Warning Text.

Warning Colour	Situation	Warning Text
Green	Normal	The water level does not exceed the threshold.The water situation is normal. No flood incident.
Orange	Surveillance	There could be a flood incident soon.People in the surveillance area should be prepared for the situation
Red	Crisis	There has occurred a flood incident in some areas. People should evacuate to a safe place.

**Table 4:** The Warning Message (Decentralized Communication Mode).

10 bytes	18 bytes	21 bytes	34 bytes	39 bytes
Date	Time	Station ID	Water Level	Warning Text

**Table 5:** Simulation Parameters.

Parameter	Value
# Data set (WaterLevel)	2400 packet
α	0.65
λ	0.1
*r*	2
η	250 min
τ	50 min
Energy used for a data transmission ($E_{t}$)	180 J/packet
Energy used in sleep mode ($E_{s}$)	0.72 J/s

**Table 6:** Percent Error of Data and Energy Improvement.

(η)	Error (%)	Energy Improvement (%)
20	0.3162	17.14
40	0.4657	17.48
60	0.5546	17.82
80	0.5466	17.65
100	0.5609	18.00

## Data Availability

Data is contained within the article.
